# Why and when citizens call for emergency help: an observational study of 211,193 medical emergency calls

**DOI:** 10.1186/s13049-015-0169-0

**Published:** 2015-11-04

**Authors:** Thea Palsgaard Møller, Annette Kjær Ersbøll, Janne Schurmann Tolstrup, Doris Østergaard, Søren Viereck, Jerry Overton, Fredrik Folke, Freddy Lippert

**Affiliations:** Emergency Medical Services Copenhagen, University of Copenhagen, Copenhagen, Denmark; National Institute of Public health, University of Southern Denmark, Copenhagen, Denmark; Danish Institute for Medical Simulation, University of Copenhagen, Copenhagen, Denmark; International Academies of Emergency Dispatch, Salt Lake City, Utah USA

**Keywords:** Emergency call, Emergency medical dispatching, Prehospital emergency care

## Abstract

**Background:**

A medical emergency call is citizens’ access to pre-hospital emergency care and ambulance services. Emergency medical dispatchers are gatekeepers to provision of pre-hospital resources and possibly hospital admissions. We explored causes for access, emergency priority levels, and temporal variation within seasons, weekdays, and time of day for emergency calls to the emergency medical dispatch center in Copenhagen in a two-year study period (December 1^st^, 2011 to November 30^th^, 2013).

**Methods:**

Descriptive analysis was performed for causes for access and emergency priority levels. A Poisson regression model was used to calculate adjusted ratio estimates for the association between seasons, weekdays, and time of day overall and stratified by emergency priority levels.

**Results:**

We analyzed 211,193 emergency calls for temporal variation. Of those, 167,635 calls were eligible for analysis of causes and emergency priority level. “Unclear problem” was the most frequent category (19 %). The five most common causes with known origin were categorized as “Wounds, fractures, minor injuries” (13 %), “Chest pain/heart disease” (11 %), “Accidents” (9 %), “Intoxication, poisoning, drug overdose” (8 %), and “Breathing difficulties” (7 %). The highest emergency priority levels (Emergency priority level A and B) were assigned in 81 % of calls. In the analysis of temporal variation, the total number of calls peaked at wintertime (26 %), Saturdays (16 %), and during daytime (39 %).

**Conclusion:**

The pattern of citizens’ contact causes fell into four overall categories: unclear problems, medical problems, intoxication and accidents. The majority of calls were urgent. The magnitude of unclear problems represents a modifiable factor and highlights the potential for further improvement of supportive dispatch priority tools or educational interventions at dispatch centers. Temporal variation was identified within seasons, weekdays and time of day and reflects both system load and disease occurrence. Data on contact patterns could be utilized in a public health perspective, benchmarking of EMS systems, and ultimately development of best practice in the area of emergency medicine.

**Electronic supplementary material:**

The online version of this article (doi:10.1186/s13049-015-0169-0) contains supplementary material, which is available to authorized users.

## Background

A medical emergency call is citizens’ access to pre-hospital emergency care and ambulance services. Emergency medical dispatchers at the emergency medical dispatch centers (EMDC) handle and prioritize the calls based on information from the callers and allocate limited pre-hospital resources. In addition they provide guidance in first aid and/or resuscitation through the telephone until arrival of the ambulance services. This is a vital point in the emergency patient trajectory [1–4] and the importance of emergency medical dispatching has been emphasized in the new guidelines for resuscitation [5]. Hence emergency medical dispatchers have an important gatekeeper function for pre-hospital emergency care and potentially hospital admissions.

There is an increasing demand for pre-hospital emergency care and ambulance services [6, 7]. Moreover emergency department overcrowding is an increasing challenge with importance for patients’ outcome [8, 9]. This underlines the significance of organizational planning of the emergency medical services (EMS) including ambulance services and EMDCs and the importance of medical dispatchers’ gatekeeper function. The majority of studies within emergency department crowding focus on in-hospital factors [8]. However, adequate triage and resource allocation in the pre-hospital setting is an important factor. Despite the potential to optimize the emergency patient flow, data on emergency medical dispatching is very sparsely reported. Emergency patients’ first contact with the system and the result of the first verbal interrogation in a nonvisual environment has not yet been described in detail and modifiable factors in emergency patients’ trajectory have rarely been explored.

In this explorative study our aim was to describe the field of emergency medical dispatching with focus on citizens’ first contact to the healthcare system through emergency calls. Specifically, we investigated 1) causes and emergency priority levels; 2) temporal variation within season, weekday and time of day.

## Methods

### Study design and setting

We conducted an analysis of emergency call data from the EMS system, Copenhagen, in a two-year study period, from December 1^st^, 2011 to November 30^th^, 2013. This period was chosen for two reasons. The EMS Copenhagen was reorganized in May 2011, implementing healthcare providers as medical dispatchers instead of police officers as call takers. Secondly, the organization’s data structure changed in December 2013. Therefore, we aimed at a study period with an unchanged organization and uniform data structure, for the purpose of the results being generalizable.

The study took place in Copenhagen, the Capital Region of Denmark with a population of 1.7 million. In Denmark, medical assistance and admission to hospitals are free of charge. In case of an emergency, there is a single emergency phone number (1-1–2) to a call center that identifies the need for police, fire or medical assistance. If the problem is medical, the caller is re-directed to the EMDC where medical dispatchers answer, process and respond to the call by activating the appropriate EMS response and delivering medical advice. The medical dispatchers are specially trained registered nurses or paramedics with experience within emergency care. Their decision-making process is supported by a criteria-based, nationwide Emergency Medical Dispatch System, Danish Index for Emergency Care version 1.2. [10], which is a validated tool for managing emergency calls for the most urgent cases of emergencies [11]. The dispatch and priority tool are a Danish adaption of the Norwegian tool originally developed by the Laerdal Foundation [12].

### Data collection and processing

The study was based on electronically registered data from a database containing all emergency calls to the EMS, Copenhagen. Danish Index for Emergency Care is integrated in the computer assisted dispatching system “Logis-CAD” [13]. In the study period we didn’t share data with hospitals.

**Fig. 1:**
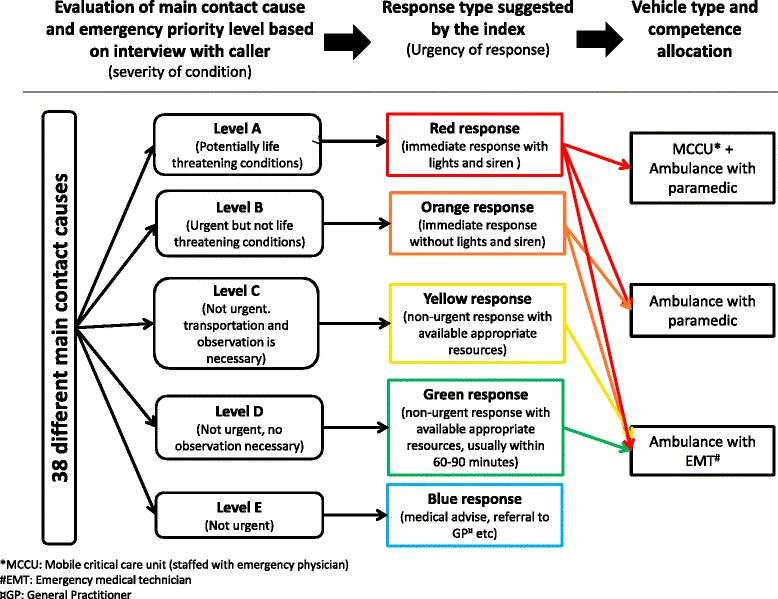
Operational workflow in the process of handling emergency calls according to Danish Index for Emergency care

The dispatch data are timestamps and information in accordance with each step of decisions in Danish Index for Emergency Care: In every emergency call, the medical dispatcher starts by asking the caller about the nature of the problem and whether the patient is awake and breathing normally. In case of suspected cardiac arrest, the dispatcher follows a guideline for dispatcher assisted cardiopulmonary resuscitation based on the European Resuscitation Council Guidelines for Resuscitation [14]. If cardiac arrest is disproved, the dispatcher follows a structured flowchart built on 38 main categories assigned for different possible causes. The flowchart guides the medical dispatcher to questions about the severity of the problem, with additional detailed questions relevant for the concrete problem. Emergency priority levels from A to E are assigned for each emergency call and refers to the urgency of the condition (level A is most urgent, level E is non-urgent). Finally, the system suggests a default response, based on the dispatchers’ decisions about main category and emergency priority level. The overall operational workflow from the emergency call/verbal interrogation to the provided response is illustrated in Fig. 1. In the EMS local guidelines it is mandatory for the medical dispatchers to follow the Danish Index for Emergency Care. However, it is possible to bypass the flowchart and only assign a response for the call, without classifying the cause, if, for example, the caller hangs up or if it is impossible to obtain adequate information from the caller. For each emergency call where the medical dispatcher follows the flowchart sufficiently, the electronic system constructs a code according to the main category of cause, decisions within each main category (depending on questions asked) and emergency priority level. Data are registered automatically according to every decision step of the flowchart. The date and time for the medical dispatchers responding to the emergency call are automatically registered for all emergency calls. The provided response is registered in 100 % of calls.

### Selection of emergency calls

**Fig. 2:**
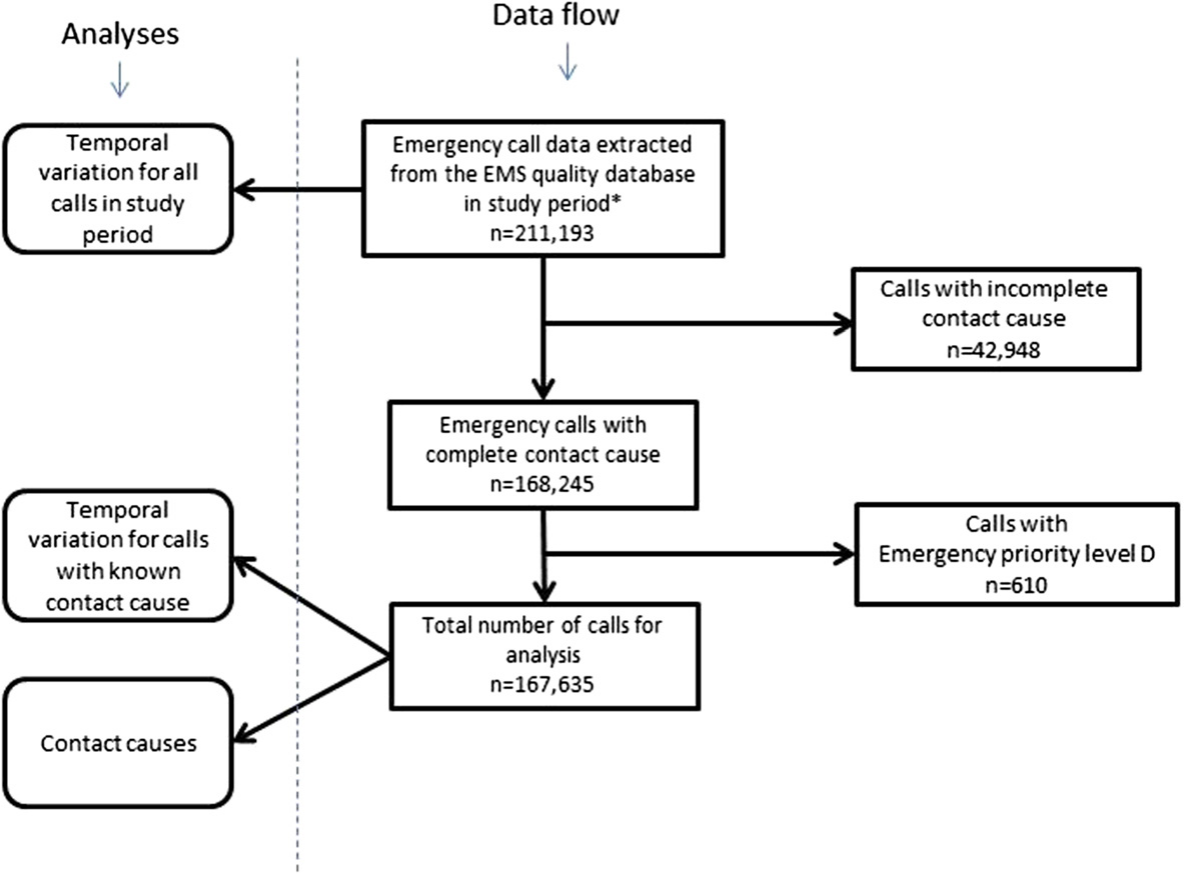
Flowchart for data collection process

We included all emergency calls in the two-year study period for analyses of temporal patterns. All 38 main categories for calls with complete coding for cause and emergency priority level were included in the descriptive analysis. We included the emergency priority levels that are considered to be an emergency condition with a medical cause and not only a need of transport. Emergency priority level D (transport only) was therefore excluded (as illustrated in data flowchart, Fig. 2).

### Derived variables

Causes and emergency priority levels were extracted from every emergency call. For temporal patterns we defined four seasons; spring (week 9–21), summer (week 22–34), autumn (week 35–47) and winter (week 48–8). For analysis of diurnal variation, we defined three time periods; daytime (07:00–14.59), evening (15:00–22:59) and nighttime (23:00–06:59). We defined two 12-month periods within the study period for the adjusted analyses, from December 1^st^, 2011 to November 30^th^, 2012 and from December 1^st^, 2012 to November 30^th^, 2013, respectively.

### Analysis

Descriptive analysis was performed by use of absolute numbers and percentages for causes and emergency priority levels. We used a Poisson regression model [15] for calculating adjusted ratio estimates for the association between seasons, weekdays and time of day and number of emergency calls as the outcome. The analyses were performed for the total number of calls in the study period, all emergency priority levels together and stratified by emergency priority level. The analyses were adjusted for differences in weeks within season and year. Pairwise comparisons between categories of significant variables (season, weekday and time of day) were performed. Pearson’s dispersion parameter was used to assess goodness-of-fit of the model. For the total amount of calls in the study period, we found inadequate model fit with a Pearson dispersion parameter at 1.92. We therefore performed sensitivity analyses, modeling the data with a model with negative binomial distribution, resulting in a dispersion parameter of 1.16 and a model with the scale fixed at 1. For the interpretation of the results, no relevant difference in the pairwise comparisons was seen. For emergency priority level C we found considerable underdispersion with Pearson dispersion parameter at 0.21. Consequently, we performed a sensitivity analysis for the model with a scaled deviance at 1, with no relevant changes in ratio estimates. The model fit was also evaluated visually by comparing the distribution of the probability of observed and fitted counts.

To address potential bias due to missing observations for cause and emergency priority level, we performed a sensitivity analysis using the provided EMS response as a proxy variable where emergency priority level was missing (See Additional file 1 for the association between assigned emergency priority levels and corresponding response provided). This analysis neither provided relevant changes in ratio estimates.

A *p*-value < 0.05 was considered statistically significant. All analyses were performed using SAS Statistical Analysis Software, version 9.3.

#### Assurances

A request for ethical approval was addressed to the Ethical Committee, Capital region of Denmark, but was not needed for this study (j.nr.: H-2-2014-FSP4). The Danish Data Protection Agency approved the conduction of the study (j.nr.: 2007-58-0015).

## Results

### Characteristics of emergency calls

In total 211,193 unique emergency calls were registered in the study period, corresponding to an emergency call incidence of 60 calls/1000 citizens/year (population in Capital Region 1,747,596 in 4^th^ quarter of 2013 [16]). Of those, 168,245 (80 %) had a complete code for cause and emergency priority level. In total 610 calls with assigned emergency priority level D were excluded, leaving 167,635 calls eligible for analysis of cause, emergency priority level and temporal variations. Data flowchart is presented in Fig. 2.

### Causes of contact to the emergency medical dispatch center through emergency calls

**Fig. 3:**
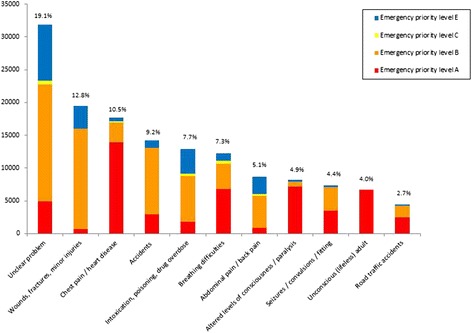
Most frequent causes (>2 % occurence).* The specific causes with corresponding percentages of all contacts (in top of bars) are listed at the x-axis. Y-axis represents the absolute numbers of contacts. The colouring of the bars represent emergency priority levels

The most common recorded cause was categorized as “Unclear problem” (19 %). The five most frequent causes with known origin counted for more than 40 % of calls in the study period and were categorized as “Wounds, fractures, minor injuries” (13 %), “Chest pain/heart disease” (11 %), “Accidents” (9 %), “Intoxication, poisoning, drug overdose” (8 %) and “breathing difficulties” (7 %). Fig. 3 illustrates causes and emergency priority levels for causes with more than 2 % occurrence. Emergency priority level A (corresponding to an emergency ambulance response with lights and siren) counted for 36 % of calls and was most commonly assigned for the causes “Chest pain/heart disease” (23 %), “Altered level of consciousness” (12 %), “Breathing difficulties” (11 %), “Unconscious (lifeless) adult” (11 %) and “Unclear problem” (8 %), respectively. Emergency priority level B (corresponding to an emergency ambulance response without lights and siren) counted for 45 % of calls and was most frequently assigned for the causes “Unclear problem” (24 %), “Wounds, fractures, minor injuries” (20 %), “Accidents” (13 %), “Intoxication, poisoning, drug overdose” (9 %) and “Abdominal pain / back pain” (7 %). Emergency priority level E (non-urgent conditions) was most commonly assigned for “Unclear problem” (33 %), “Intoxication, poisoning, drug overdose” (15 %), “Wounds, fractures, minor injuries” (13 %), “Abdominal pain/back pain” (10 %) and “Psychiatry/suicide” (5 %). In Additional file 2, the frequency and percentages for all causes in total and for each emergency priority level in the study period is presented.

### Temporal variations for emergency calls

Table 1 shows total numbers and percentages of emergency calls overall and stratified by season, weekday and time of day according to emergency priority levels for all calls in study period, for all calls with a complete registered cause and calls within each emergency priority level.

**Table 1:** Total numbers and percentages of emergency calls overall and stratified by season, weekday and time of day according to emergency priority levels

	Emergency priority level^b^					
	A	B	C	E	Total, known contact cause	Total in study period^a^
Total, n(%)	60,746 (100)	75,555 (100)	5,550 (100)	25,784 (100)	167,635 (100)	211,193 (100)
Variable						
*Season* ^c^						
Winter, n (%)	15,624 (25.7)	19,096 (25.2)	1,119 (20.2)	6,365 (24.7)	42,204 (25.2)	53,808 (25.5)
Spring, n (%)	15,091 (24.8)	18,378 (24.3)	1,416 (25.5)	5,922 (23.0)	40,807 (24.3)	51,833 (24.5)
Summer, n (%)	15,326 (25.2)	19,181 (25.4)	1,586 (28.6)	6,669 (25.9)	42,762 (25.5)	53,167 (25.2)
Autumn, n (%)	14,705 (24.2)	18,900 (25.0)	1,429 (25.8)	6,828 (26.5)	41,862 (25.0)	52,385 (24.8)
*Weekday*						
Monday, n (%)	8,982 (14.8)	10,457 (13.8)	717 (12.9)	3,120 (12.1)	23,276 (13.9)	29,157 (13.8)
Tuesday, n (%)	8,762 (14.4)	10,352 (13.7)	733 (13.2)	3,057 (11.9)	22,904 (13.7)	28,564 (13.5)
Wednesday, n(%)	8,612 (14.2)	10,338 (13.7)	753 (13.6)	3,152 (12.2)	22,855 (13.6)	28,401 (13.5)
Thursday, n (%)	8,794 (14.5)	10,813 (14.3)	763 (13.8)	3,204 (12.4)	23,574 (14.1)	29,471 (14.0)
Friday, n (%)	8,926 (14.7)	11,343 (15.0)	859 (15.5)	3,900 (15.1)	25,028 (14.9)	31,320 (14.8)
Saturday, n (%)	8,524 (14.0)	11,634 (15.4)	922 (16.6)	4,825 (18.7)	25,905 (15.5)	33,142 (15.7)
Sunday, n (%)	8,146 (13.4)	10,618 (14.1)	803 (14.5)	4,526 (17.6)	24,093 (14.4)	31,138 (14.7)
*Time of day* ^d^						
Night, n (%)	11,604 (19,1)	16,314 (21,6)	1,123 (20.2)	8,430 (32.7)	37,471 (22.4)	49,104 (23.3)
Day, n (%)	26,433 (43,5)	30,880 (40,9)	2,363 (42.6)	7,660 (29.7)	67,336 (40.2)	82,242 (38.9)
Evening, n (%)	22,709 (37,4)	28,361 37,5)	2,064 (37.2)	9,694 (37.6)	62,828 (37.5)	79,847 (37.8)

^a^Data from a two year study period (1/12-2011-30/11-2013)

^b^Emergency priority level A = red response (potentially life threatening). Emergency priority level B = orange response (acute, but not life threatening). Emergency priority level C = yellow response (not acute, but transportation and observation in ambulance is necessary). Emergency priority level E = Blue response (Advise, recommendation, referral to general practitioner etc

^c^Seasons are defined as winter (week 48–8), spring (week 9–21), summer (week 22–34) and autumn (week 35–47)

^d^The day is divided into three periods: nighttime (23:00–06:59), daytime (07:00–14.59) and evening (15:00–22:59)

Temporal variation in emergency calls

The highest number of calls occurred in winter (used as reference in the pairwise comparisons) and the lowest number in spring (ratio estimate 0.97, CI 0.96–0.98). The number of calls was highest in weekends (Fridays, Saturdays and Sundays) compared with working days. The peak number of calls was seen on Saturdays (ratio estimate 1.14, CI 1.12–1.15) and the lowest number on Wednesdays (ratio estimate 0.98, CI 0.96–0.99) compared to Mondays. The number of calls was highest in daytime (ratio estimate 1.67, CI 1.65–1.69), compared to evening (ratio estimate 1.64, CI 1.62–1.66) and nighttime (used as a reference).

**Fig. 4:**
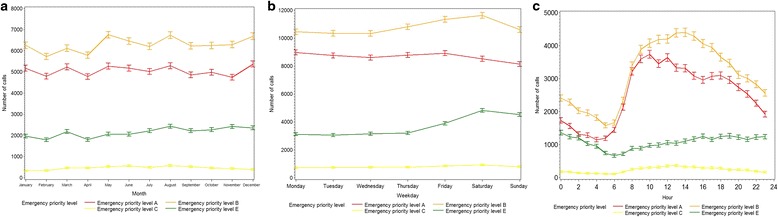
Monthly, weekday and diurnal variation of emergency calls with complete registered cause by emergency priority level (number of calls, 95 % confidence interval). **a** Monthly variation. **b** Weekday variation. **c** Diurnal variation

For weekday variation, emergency priority level A showed a different trend than the rest of the emergency priority levels, with peak number of calls on Mondays and the lowest number on Sundays. Emergency priority level E showed the greatest weekday variation with weekends being significantly different than the other days, seen by ratio estimates of 1.13, CI 1.08–1.19 at Fridays, 1.30, CI 1.24–1.36 at Saturdays and 1.25, CI 1.20–1.31 at Sundays. The diurnal variation differed for emergency priority level E, with a peak in calls in the evening compared to nighttime and daytime. Figure 4 illustrates, monthly, weekday and diurnal variation according to each emergency priority level.

### Diurnal variation for common causes

**Fig. 5:**
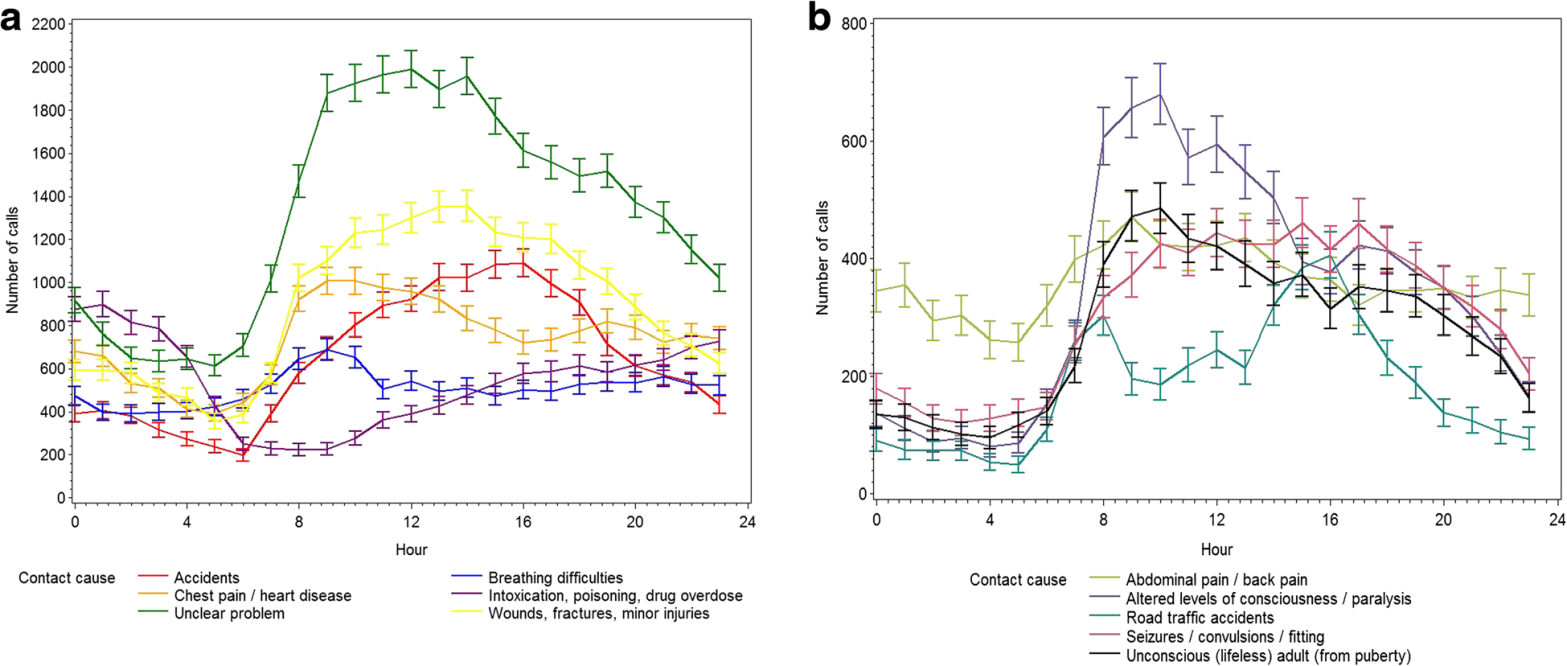
Diurnal variation for most common causes of emergency calls in study period. **a** contact2 counting for > 6 % of total number of calls. **b** contacts counting for 2–6 % of total number of calls

Figure 5 is a graphical illustration of diurnal variation for calls with a complete cause registered with more than 2 % occurrence. The figure shows different trends for different causes. “Chest pain/heart disease”, “Unconscious (lifeless) adult” and “Altered level of consciousness” has two peaks during the day; at morning and at evening. “Wounds, fractures, minor injuries” and “Accidents“peaks at noon, whereas “Intoxication, poisoning, drug overdose” peaks at nighttime. For”Road traffic accidents”, the trend is maximum number of calls in the morning (8 AM) and afternoon (4 PM).

## Discus*s*ion

In this explorative study of 211, 193 emergency calls from a two-year study period, we aimed at describing causes, emergency priority levels and temporal patterns within season, weekday and time of day, in emergency calls to a large emergency medical dispatch center in Denmark. We found an emergency call incidence of 60/1000 citizens/year. This number may reflect the magnitude of need for emergency care in the population. Due to the lack of similar studies, it is unknown whether the incidence reflects a high or low use of emergency calls. The incidence might vary in different countries and settings depending on culture, educational level or organization of out-of-hour services. Comparison with other organizations would be valuable in terms of identifying opportunities for efficiency gains.

Interestingly, the 5 most common causes with known origin counted for more than 40 % of calls and were categorized as “Wounds, fractures, minor injuries”, “Chest pain/heart disease”, “Accidents”, “Intoxication, poisoning, drug overdose”, and “Breathing difficulties”. The highest emergency priority level (level A) counted for 36 % of calls and was most commonly assigned for the causes “Chest pain/heart disease”, “Altered level of consciousness”, “Breathing difficulties”, “Unconscious (lifeless) adult” and “Unclear problem.” We identified the highest incidence of emergency calls in the winter (26 %), weekends (45 %) and during daytime (39 %) for the total number of calls.

### Most common causes

The pattern of the citizens’ causes and thereby need for emergency care could be categorized into four overall categories: unclear problems, medical problems, intoxication and accidents. This pattern is comparable with data reported from other dispatch centers [11, 17–19]. Knowledge about the most common causes can be used in various levels; it provides an opportunity for disease surveillance and monitoring, important in a public health perspective like suggested previously [20, 21]. Moreover, the organizations can learn where to prioritize their resources and target their education and quality assurance at their dispatch centers.

Recognition of time critical diseases is crucial for early treatment, timely response and patients’ outcome [1–4]. In that sense, the magnitude of unclear problems is likely to have clinical implications. “Unclear problem” is usually assigned because the medical dispatchers don’t know the exact medical cause. However an assessment of the urgency of the response is still performed based on the interview with the caller. Our findings are comparable with other studies from dispatch centers using the similar criteria based priority tools [11, 17]. When looking at the emergency priority levels assigned for the unclear problem category, 15 % of those calls had the highest urgency (emergency priority level A). This might implicate potential missed opportunities for correct pre-arrival instructions to the caller and proper and timely emergency care. This is supported by a recent study looking at potentially preventable same day deaths following an emergency call, identifying 18 potentially preventable deaths of which 7 was categorized as “Unclear problem” [22].

The great proportion of unclear problem categorization could reflect a weakness in the medical dispatching system and a modifiable factor worthwhile exploring in future studies. For instance, a comparison study between organizations, with discussion of underlying reasons and improvement of systems would be valuable, like previously done in a study comparing recognition of out-of-hospital cardiac arrest in two commonly used dispatch concepts Medical Priority Dispatch System and Criteria Based Dispatch [23]. In a study of emergency call types registered in a Medical Priority Dispatch System, the category “general illness” represented 17 % of calls [24]. General illness may represent some of the unclear problems in our study and adding a general illness category to the Danish Index for Emergency care might be beneficial.

Finally the magnitude of unclear problems illustrates the complexity of dispatchers’ handling and triaging of emergency calls without any opportunity for an objective assessment of the patient or evaluation of the callers’ surroundings, besides using communication skills. Language barriers, callers’ emotional state, callers’ ability to describe the situation or the medical dispatchers’ skills in asking proper questions may affect the situation [2, 25, 26]. Therefore, it is important to design and maintain systems that support the medical dispatchers’ decision making process along with offering them sufficient education and communication training.

### Causes within the most and the least urgent emergency priority levels

Within emergency priority level A (the most urgent), the four most common causes were “Chest pain/heart disease”, “Altered level of consciousness”, “Breathing difficulties” and “Unconscious (lifeless) adult”. These causes are all time critical and in accordance with the four most common causes of deaths in Denmark, which is ischemic heart disease (IHD), lung cancer, stroke, and chronic obstructive pulmonary disease (COPD) [27]. Emergency priority level E (non-urgent conditions resulting in a medical advice) was most commonly assigned for “Unclear problem”, “Intoxication, poisoning, drug overdose”, “Wounds, fractures, minor injuries”, “Abdominal pain/back pain” and “Psychiatry/suicide”. This result is in line with a study by Marks at al who investigated non-transported ambulance services [28].

The differences in patterns in the most urgent vs. the non-urgent calls are interesting from an organizational point of view. The medical dispatchers have to deal with a wide range of medical diseases and injuries and depending on the urgency of the condition or situation, they must adapt their guidance and medical advice to each individual situation. At the same time they have the responsibility of deciding whether or not to send an ambulance and prioritizing the EMS resources. Finally, the task of talking to citizens with different demographic, social and ethnical background, adds to the challenges in handling emergency calls, as illustrated in a recent study that identified age and gender being associated with the EMS priority [29]. Identifying which calls warrants lights and siren ambulance response is a central role in emergency medical dispatch practice and crucial for the gatekeeper function of the dispatch centers. Methodologies for optimization of this work process are rarely reported. However, using local retrospective data in identifying the optimal mix of response specificity and system sensitivity might be one opportunity, like suggested in a previous study [30].

### Temporal variation in emergency calls

Our study showed temporal variations in emergency calls both within seasons, weekdays and time of day. Exact reasons for the variations are unknown but the results may generate hypotheses and have implications. Our results from the analyses of weekday and diurnal variation are clinically important and similar to those from a large recent study investigating diurnal and weekday variation in EMS demand [31]. However, they mainly focused on ambulance services, using assessment data from paramedics and not emergency medical dispatch coding data.

We included all contacts to the EMS including emergency calls where a medical advice and no ambulance response were provided. The majority of the most urgent calls were seen in daytime and the non-urgent calls peaked in evenings. The pattern for non-urgent calls could reflect the opening hours for general practitioners. This leads to the question of which competences and skills should be represented at the EMDC’s and when. The individual EMS organizations must take into account the different workload and case mix at the EMDC’s depending on the time of day and day of the week.

We graphically demonstrated the diurnal variation for the most common causes. Interestingly, the different diseases or conditions showed different diurnal patterns, which is similar to results from a study looking at peaks in emergency calls within different groups of conditions [32]. The diurnal variation within different causes of contacts may predict workload at the EMDC, but also disease occurrence. For out-of-hospital cardiac arrest, diurnal variation has been identified with lowest incidence at nighttime [33, 34], and interestingly the lowest 30-day survival [35]. Several categories of diseases, both chronic and emergency, also show diurnal patterns [36]. Moreover, the diurnal patterns might reflect citizens’ behavior. The high occurrence of road traffic accidents in the morning and afternoon reflects rush hours, and intoxication peaking at nighttime might be a metropolitan phenomenon. These events could be used in planning and monitoring health care and infrastructure interventions and in combination with geographical data, there would be an additional unique opportunity to target those interventions in the community.

#### Study limitations

Our study has some limitations. Our aim was to investigate contact patterns to an emergency medical call-center in terms of temporal variations and causes. The causes and emergency priority levels were extracted from a specific variable from the EMS database. This variable had 20 % missing values. However it was the only variable with information about the causes and urgency of the condition of the caller. For the analyses of temporal variation, we addressed the problem of missing values with a sensitivity analysis with the response type as a proxy variable, in cases with missing cause and emergency priority level, and found no changes in the pairwise comparisons. Data in this study were all registered electronically, but the specific variable concerning cause and emergency priority level, reflects medical dispatchers’ decisions during an emergency call according to the flowchart in their supportive priority tool. This may introduce classification bias. Finally, the study was conducted in Copenhagen, Denmark, and the results may reflect a pattern for a capital. This might decrease the generalizability of the identified contact patterns in a national and international perspective. Importantly, focus on transparency and uniform reporting of EMS and emergency medical dispatch data is increasing worldwide [37, 38].

## Conclusion

The pattern of citizens’ contact causes and thereby need for emergency care fell into four overall categories: unclear problems, medical problems, intoxication and accidents. The five most common causes with known origin counted for more than 40 % of all calls and the vast majority of emergency calls were assigned the most acute emergency priority levels. Temporal variation was identified within seasons, weekdays and time of day and reflects both the system load and disease occurrence. The magnitude of unclear problems represents a modifiable factor and highlights the potential for further improvement of supportive dispatch priority tools or educational interventions at dispatch centers. Data on contact patterns could be utilized in a public health perspective, benchmarking of EMS systems, and ultimately the development of best practice in the area of emergency medicine.

## Additional files

Additional file 1:
**Association between emergency priority levels and response types, shown in bars and frequencies.** (JPEG 52 kb) Additional file 2:
**Main Criteria by Emergency priority level in study period 1.12.2011-30.11.2013.** (DOCX 18 kb)

## References

[1] Berdowski J, Beekhuis F, Zwinderman AH, Tijssen JG, Koster RW (2009). Importance of the first link: description and recognition of an out-of-hospital cardiac arrest in an emergency call. Circulation.

[2] Lewis M, Stubbs BA, Eisenberg MS (2013). Dispatcher-assisted cardiopulmonary resuscitation: time to identify cardiac arrest and deliver chest compression instructions. Circulation.

[3] Herlitz J, Wireklintsundström B, Bång A, Berglund A, Svensson L, Blomstrand C (2010). Early identification and delay to treatment in myocardial infarction and stroke: differences and similarities. Scand J Trauma Resusc Emerg Med.

[4] Buck BH, Starkman S, Eckstein M, Kidwell CS, Haines J, Huang R (2009). Dispatcher recognition of stroke using the National Academy Medical Priority Dispatch System. Stroke J Cereb Circ.

[5] Monsieursa KG, Nolan JP, Bossaerte LL, Greiff R, Maconochieh IK, Nikolaoui NI (2015). European Resuscitation Council Guidelines for Resuscitation 2015 Section 1. Executive summary. Resuscitation.

[6] Lowthian JA, Cameron PA, Stoelwinder JU, Curtis A, Currell A, Cooke MW (2011). Increasing utilisation of emergency ambulances. Aust Health Rev Publ Aust Hosp Assoc.

[7] Pittet V, Burnand B, Yersin B, Carron PN (2014). Trends of pre-hospital emergency medical services activity over 10 years: a population-based registry analysis. BMC Health Serv Res.

[8] Bernstein SL, Aronsky D, Duseja R, Epstein S, Handel D, Hwang U (2009). The effect of emergency department crowding on clinically oriented outcomes. Acad Emerg Med.

[9] Sun BC, Hsia RY, Weiss RE, Zingmond D, Liang LJ, Han W (2013). Effect of emergency department crowding on outcomes of admitted patients. Ann Emerg Med.

[10] Dansk Indeks - Dansk Indeks version 1.2_010212.pdf [Internet]. [cited 2015 Mar 6]. Available from: https://www.regionh.dk/om-region-hovedstaden/Den-Praehospitale-Virksomhed/Akutberedskabets-organisation/112-AMK-Vagtcentralen/Documents/Dansk%20Indeks%20version%201.5%20-%20landsudgaven%20(enkeltsider).pdf

[11] Andersen MS, Johnsen SP, Sørensen JN, Jepsen SB, Hansen JB, Christensen EF (2013). Implementing a nationwide criteria-based emergency medical dispatch system: A register-based follow-up study. Scand J Trauma Resusc Emerg Med.

[12] Norsk indeks for medisinsk nødhjelp.pdf [Internet]. [cited 2015 May 4]. Available from: http://www.helse-midt.no/ftp/stolav/eqspublic/pasientforlop/docs/doc_26316/attachments/Norsk%20indeks%20for%20medisinsk%20n%C3%B8dhjelp.pdf.

[13] Logis Solutions A/S [Internet]. [cited 2015 May 4]. Available from: http://www.logiscad.com/.

[14] Nolan JP, Hazinski MF, Billi JE, Boettiger BW, Bossaert L, de Caen AR (2010). Part 1: Executive summary: 2010 International Consensus on Cardiopulmonary Resuscitation and Emergency Cardiovascular Care Science With Treatment Recommendations. Resuscitation.

[15] Coxe S, West SG, Aiken LS (2009). The analysis of count data: a gentle introduction to poisson regression and its alternatives. J Pers Assess.

[16] Statistikbanken [Internet]. [cited 2015 Mar 4]. Available from: http://www.statistikbanken.dk/statbank5a/default.asp?w=1366.

[17] Ellensen EN, Hunskaar S, Wisborg T, Zakariassen E (2014). Variations in contact patterns and dispatch guideline adherence between Norwegian emergency medical communication centres - a cross-sectional study. Scand J Trauma Resusc Emerg Med.

[18] Zakariassen E, Burman RA, Hunskaar S (2010). The epidemiology of medical emergency contacts outside hospitals in Norway--a prospective population based study. Scand J Trauma Resusc Emerg Med.

[19] Gaffney P, Crane S, Johnson G, Playforth M (2001). An analysis of calls referred to the emergency 999 service by NHS Direct. Emerg Med J EMJ.

[20] Krafft T, García Castrillo-Riesgo L, Edwards S, Fischer M, Overton J, Robertson-Steel I (2003). European Emergency Data Project (EED Project): EMS data-based health surveillance system. Eur J Public Health.

[21] Ziemann A, Rosenkötter N, Garcia-Castrillo Riesgo L, Schrell S, Kauhl B, Vergeiner G (2014). A concept for routine emergency-care data-based syndromic surveillance in Europe. Epidemiol Infect.

[22] Andersen MS, Johnsen SP, Hansen AE, Skjaerseth E, Hansen CM, Sørensen JN (2014). Preventable deaths following emergency medical dispatch - an audit study. Scand J Trauma Resusc Emerg Med.

[23] Hardeland C, Olasveengen TM, Lawrence R, Garrison D, Lorem T, Farstad G (2014). Comparison of Medical Priority Dispatch (MPD) and Criteria Based Dispatch (CBD) relating to cardiac arrest calls. Resuscitation.

[24] Munjal KG, Silverman RA, Freese J, Braun JD, Kaufman BJ, Isaacs D (2011). Utilization of emergency medical services in a large urban area: description of call types and temporal trends. Prehosp Emerg Care.

[25] Ornato JP (2009). Science of emergency medical dispatch. Circulation.

[26] Meischke HW, Calhoun RE, Yip MP, Tu SP, Painter IS (2013). The effect of language barriers on dispatching EMS response. Prehospital Emerg Care.

[27] GBD 2013 Mortality and Causes of Death Collaborators (2015). Global, regional, and national age- sex specific all-cause and cause-specific mortality for 240 causes of death, 1990–2013: a systematic analysis for the Global Burden of Disease Study 2013. Lancet.

[28] Marks PJ, Daniel TD, Afolabi O, Spiers G, Nguyen-Van-Tam JS (2002). Emergency (999) calls to the ambulance service that do not result in the patient being transported to hospital: an epidemiological study. Emerg Med J EMJ.

[29] Rawshani A, Larsson A2, Gelang C2, Lindqvist J3, Gellerstedt M4, Bång A (2014). et al. Characteristics and outcome among patients who dial for the EMS due to chest pain. Int J Cardiol.

[30] Craig AM, Verbeek PR, Schwartz B (2010). Evidence-based optimization of urban firefighter first response to emergency medical services 9-1-1 incidents. Prehosp Emerg Care Marts.

[31] Cantwell K, Morgans A, Smith K, Livingston M, Spelman T, Dietze P, et al. Time of Day and Day of Week Trends in EMS Demand. Prehospital Emerg Care. 2015; doi:10.3109/10903127.2014.995843 [Epub ahead of print]. 10.3109/10903127.2014.995843 25664379

[32] Manfredini R, La Cecilia O, Boari B, Steliu J, Michelinidagger V, Carlidagger P (2002). Circadian pattern of emergency calls: implications for ED organization. Am J Emerg Med.

[33] Herlitz J, Eek M, Holmberg M, Holmberg S (2002). Diurnal, weekly and seasonal rhythm of out of hospital cardiac arrest in Sweden. Resuscitation.

[34] Nakanishi N, Nishizawa S, Kitamura Y, Nakamura T, Matsumuro A, Sawada T (2011). Circadian, weekly, and seasonal mortality variations in out-of-hospital cardiac arrest in Japan: analysis from AMI-Kyoto Multicenter Risk Study database. Am J Emerg Med.

[35] Karlsson LI, Wissenberg M, Fosbøl EL, Hansen CM, Lippert FK, Bagai A (2014). Diurnal variations in incidence and outcome of out-of-hospital cardiac arrest including prior comorbidity and pharmacotherapy: a nationwide study in Denmark. Resuscitation.

[36] Smolensky MH, Portaluppi F, Manfredini R, Hermida RC, Tiseo R, Sackett-Lundeen LL (2014). Diurnal and twenty-four hour patterning of human diseases: acute and chronic common and uncommon medical conditions. Sleep Med Rev.

[37] Castrén M, Bohm K, Kvam AM, Bovim E, Christensen EF, Steen-Hansen JE (2011). Reporting of data from out-of-hospital cardiac arrest has to involve emergency medical dispatching--taking the recommendations on reporting OHCA the Utstein style a step further. Resuscitation.

[38] Perkins GD, Jacobs IG, Nadkarni VM, Berg RA, Bhanji F, Biarent D, et al. Cardiac arrest and cardiopulmonary resuscitation outcome reports: Update of the Utstein resuscitation registry templates for out-of-hospital cardiac arrest. Resuscitation. 2014; doi:10.1016/j.resuscitation.2014.11.002. [Epub ahead of print].

