# Supplementary figures and images for: Why and when citizens call for emergency help: an observational study of 211,193 medical emergency calls
Source: Scand J Trauma Resusc Emerg Med. 2015 Nov 4;23:88. doi: 10.1186/s13049-015-0169-0 (PMC4632270; doi:10.1186/s13049-015-0169-0)

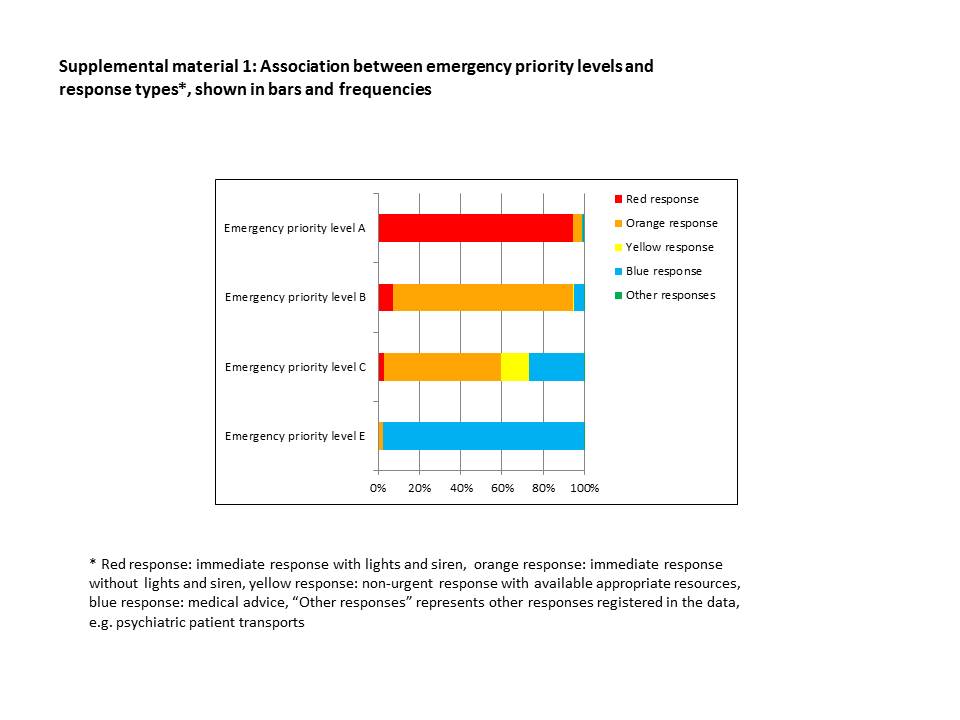

Supplement: Additional file 1: — Association between emergency priority levels and response types, shown in bars and frequencies. (JPEG 52 kb) [file 13049_2015_169_MOESM1_ESM.jpg]
