# Supplementary material for: Why and when citizens call for emergency help: an observational study of 211,193 medical emergency calls
Source: Scand J Trauma Resusc Emerg Med. 2015 Nov 4;23:88. doi: 10.1186/s13049-015-0169-0 (PMC4632270; doi:10.1186/s13049-015-0169-0)
Supplement: Additional file 2: — Main Criteria by Emergency priority level in study period 1.12.2011-30.11.2013. (DOCX 18 kb) [file 13049_2015_169_MOESM2_ESM.docx]

**Supplemental material 2. Main Criteria by Emergency priority level in study period 1.12.2011-30.11.2013**

| **Main Criteria** | **Total (%)** | **Level A (%)** | **Level B (%)** | **Level C (%)** | **Level E (%)** |
| --- | --- | --- | --- | --- | --- |
| 1. Unclear problem | 32,123 (19.09) | 4,957 (8.16) | 17,832 (23.60) | 567 (10.22) | 8481 (32.89) |
| 2. Wounds, fractures, minor injuries | 21,573 (12.82) | 698 (1.15) | 15,332 (20.29) | 1,902 (34.27) | 3437 (13.33) |
| 3. Chest pain / heart disease | 17,658 (10.50) | 13,975 (23.01) | 2,991 (3.96) | 178 (3.21) | 514 (1.99) |
| 4. Accidents | 15,554 (9.24) | 2,991 (4.92) | 10,111 (13.38) | 1,272 (22.92) | 1097 (4.25) |
| 5. Intoxication, poisoning, drug overdose | 12,903 (7.67) | 1,812 (2.98) | 6,964 (9.22) | 329 (5.93) | 3798 (14.73) |
| 6. Breathing difficulties | 12,206 (7.25) | 6,849 (11.27) | 3,831(5.07) | 407 (7.33) | 1119 (4.34) |
| 7. Abdominal pain / back pain | 8,644 (5.14) | 860 (1.42) | 4,918 (6.51) | 311 (5.60) | 2555 (9.91) |
| 8. Altered levels of consciousness / paralysis | 8,225 (4.89) | 7,185 (11.83) | 712 (0.94) | 81 (1.46) | 247 (0.96) |
| 9. Seizures / convulsions / fitting | 7,385 (4.39) | 3,521 (5.80) | 3,601 (4.77) | 0 (0.00) | 263 (1.02) |
| 10. Unconscious (lifeless) adult (from puberty) | 6,696 (3.98) | 6,696 (11.02) | 0 (0.00) | 0 (0.00) | 0 (0.00) |
| 11. Road traffic accidents | 4,459 (2.65) | 2,522 (4.15) | 1,729 (2.29) | 0 (0.00) | 198 (0.77) |
| 12. Bleeding (non-trauma) | 3,028 (1.80) | 1,503 (2.47) | 1,121 (1.48) | 121 (2.18) | 283 (1.10) |
| 13. Psychiatry / suicide | 2,858 (1.70) | 639 (1.05) | 983 (1.30) | 0 (0.00) | 1236 (4.79) |
| 14. Diabetes | 2,780 (1.65) | 936 (1.54) | 1,651 (2.19) | 91 (1.64) | 102 (0.40) |
| 15. Allergic reaction | 1,523 (0.91) | 1,034 (1.70) | 361 (0.48) | 0 (0.00) | 128 (0.50) |
| 16. Violence / abuse | 1,329 (0.79) | 351 (0.58) | 817 (1.08) | 0 (0.00) | 161 (0.62) |
| 17. Sick children | 1,171 (0.70) | 599 (0.99) | 229 (0.30) | 0 (0.00) | 343 (1.33) |
| 18. Ear, nose and throat | 1,118 (0.66) | 153 (0.25) | 684 (0.91) | 13 (0.23) | 268 (1.04) |
| 19. Gynecology / pregnancy | 1,084 (0.64) | 499 (0.82) | 228 (0.30) | 113 (2.04) | 244 (0.95) |
| 20. Urinary tract | 961 (0.57) | 18 (0.03) | 651 (0.86) | 8 (0.14) | 284 (1.10) |
| 21. Headache | 857 (0.51) | 496 (0.82) | 15 (0.02) | 76 (1.37) | 270 (1.05) |
| 22. Possible death / cot death | 727 (0.43) | 651 (1.07) | 76 (0.10) | 0 (0.00) | 0 (0.00) |
| 23. Burns / electrical injuries | 643 (0.38) | 279 (0.46) | 262 (0.35) | 0 (0.00) | 102 (0.40) |
| 24. Choking/foreign body in throat | 519 (0.31) | 455 (0.75) | 22 (0.03) | 0 (0.00) | 42 (0.16) |
| 25. Fever | 495 (0.29) | 173 (0.28) | 74 (0.10) | 0 (0.00) | 248 (0.96) |
| 26. The eyes | 354 (0.21) | 81 (0.13) | 131 (0.17) | 17 (0.31) | 125 (0.48) |
| 27. Unconscious (lifeless) child (pre puberty) | 319 (0.19) | 319 (0.53) | 0 (0.00) | 0 (0.00) | 0 (0.00) |
| 28. Transport reservations | 319 (0.19) | 113 (0.19) | 68 (0.09) | 4 (0.07) | 130 (0.50) |
| 29. Childbirth | 248 (0.15) | 184 (0.30) | 9 (0.01) | 0 (0.00) | 32 (0.12) |
| 30. Animal bites / insect stings | 109 (0.06) | 50 (0.08) | 15 (0.02) | 17 (0.31) | 27 (0.10) |
| 31. Chemicals / gasses | 108 (0.06) | 31 (0.05) | 45 (0.06) | 5 (0.09) | 27 (0.10) |
| 32. Hypothermia / hyperthermia | 79 (0.05) | 24 (0.04) | 49 (0.06) | 0 (0.00) | 6 (0.02) |
| 33. Drowning | 65 (0.04) | 47 (0.08) | 17 (0.02) | 0 (0.00) | 1 (0.00) |
| 34. Poisoning in children | 50 (0.03) | 15 (0.02) | 15 (0.02) | 20 (0.36) | 0 (0.00) |
| 35. Skin complaints and rash | 37 (0.02) | 18 (0.03) | 0 (0.00) | 3 (0.05) | 16 (0.06) |
| 36. Psychiatric emergency service | 27 (0.02) | 2 (0.00) | 10 (0.01) | 15 (0.27) | 0 (0.00) |
| 37. Scuba-diving accidents | 7 (0.00) | 7 (0.01) | 0 (0.00) | 0 (0.00) | 0 (0.00) |
| 38. Major Incidents | 4 (0.00) | 3 (0.00) | 1 (0.00) | 0 (0.00) | 0 (0.00) |
| **Total** | **167,635 (100)** | **60,746 (100)** | **75,555 (100)** | **5,550 (100)** | **25,784 (100)** |
